# The Role of miRNAs in Chemotherapy-Induced Cardiotoxicity

**DOI:** 10.3390/biomedicines13102331

**Published:** 2025-09-24

**Authors:** Maria Anastasiou, Evangelos Oikonomou, Panagiotis Theofilis, Maria Gazouli, Amanda Psyrri, Flora Zagouri, Gerasimos Siasos, Dimitrios Tousoulis

**Affiliations:** 1 Section of Oncology, 2nd Propaedeutic Department of Internal Medicine and Clinical Research Institute, Attikon University Hospital, National and Kapodistrian University of Athens, 12462 Athens, Greece; 2 3rd Department of Cardiology, Sotiria Chest Disease Hospital, Medical School, National and Kapodistrian University of Athens, 11527 Athens, Greece; boikono@gmail.com (E.O.);; 3 1st Department of Cardiology, “Hippokration” Athens General Hospital, Medical School, University of Athens, 11527 Athens, Greece; 4 Laboratory of Biology, Department of Basic Medical Sciences, Medical School, National and Kapodistrian University of Athens, 11527 Athens, Greece; 5 Department of Clinical Therapeutics, Alexandra Hospital, Medical School, National and Kapodistrian University of Athens, 11528 Athens, Greece

**Keywords:** microRNAs, chemotherapy-induced cardiotoxicity, biomarker, anthracycline

## Abstract

Cardiotoxicity is one of the most important adverse events of chemotherapy regimens, especially of anthracyclines. Different mechanisms are associated with chemotherapy-related cardiac dysfunction (CTRCD): oxidative stress, mitochondrial dysfunction, inhibition of topoisomerase 2 beta, abnormal iron metabolism, apoptosis, and fibrosis. Even after years of investigation, the early detection and prevention of cardiac impairment after chemotherapy through biomarkers remains an unmet need. The differential expression of microRNAs (miRs) in plasma at different timepoints (baseline, stable intervals during and at the end of chemotherapy) has been associated with CTRCD. Namely, some miRs, such as let-7, miR-29 and miR-30 family, miR-1 clusters, miR-34a, miR-126, miR-130a, miR-140, miR-320a, and miR-499, could play prognostic and/or diagnostic roles in CTRCD. Key miRs involved in apoptosis and oxidative stress include miR-1, miR-21, miR-30 and miR-130a, while let-7 family, miR-34a, miR-29b and miR-499 are associated with fibrosis and extracellular matrix remodeling. Additionally, mitochondrial function is regulated by miR-30, miR-130a and miR-499. Expanding its role, miR-130a could act as a therapeutic agent of CTRCD through its inhibition. This narrative review focuses on the current understanding of miRs’ involvement in CTRCD pathophysiology, summarizes the evidence linking miRs with cardiotoxicity risk, and explores the potential of miRs as biomarkers and therapeutic targets to improve early detection, risk stratification, and management of CTRCD.

## 1. Introduction

As survival rates of cancer patients have improved, the medical community increasingly focuses on managing adverse effects of anticancer therapies to enhance cancer survivors’ quality of life. One significant long-term adverse event is chemotherapy-related cardiac dysfunction (CTRCD), as antineoplastic agents such as anthracyclines and trastuzumab, while effective against cancer, can also cause cardiac impairment. Identifying prognostic factors of chemotherapy-induced cardiotoxicity is crucial to detect patients likely to develop CTRCD early, thereby preventing irreversible cardiac damage. Cardioprotective therapeutic strategies have been developed to mitigate this risk, including pharmacological interventions such as beta-blockers, angiotensin-converting enzyme inhibitors, angiotensin receptor blockers, mineralocorticoid receptor antagonists, statins, and dexrazoxane [1,2,3].

MicroRNAs (miRs) are small (18–25 nucleotides) non-coding, single-stranded RNA parts that are stable in the circulation, and some of them are involved in molecular mechanisms and pathways associated with CTRCD [4]. For this reason, they may serve as candidate biomarkers with prognostic value for the early detection of CTRCD. Most of the trials are focused on anthracycline-induced cardiotoxicity and the mechanisms that lead to cardiac injury and impairment. Anthracyclines are used as part of multimodal treatment against different types of cancer, such as breast cancer and sarcomas. Although they show a cardiotoxicity incidence of 10–16%, it has also been reported up to 57% of asymptomatic cardiac impairment [5,6,7]. In this narrative review, we examine the role of miRs in the molecular mechanisms underlying CTRCD, summarize clinical studies linking circulating miR profiles to cardiotoxicity risk, and explore their potential as biomarkers and therapeutic targets for improving early detection, risk stratification, and management of CTRCD.

## 2. Methods

We conducted a comprehensive literature search to identify studies investigating the role of miRs in CTRCD. Searches were performed in PubMed and Google Scholar databases through May 2025. The search strategy combined relevant keywords such as “chemotherapy-induced cardiotoxicity”, “CTRCD”, “cardiac dysfunction”, “microRNAs”, “miR biomarkers”, and “anthracyclines”.

## 3. Mechanisms of CTRCD and miRNAs’ Correlation

The chemotherapy regimens most associated with CTRCD are anthracyclines, particularly doxorubicin. MiRs play a key regulatory role in gene expression, influencing several pathophysiological mechanisms involved in CTRCD. These mechanisms and their correlations with specific miRs are summarized in Table 1.

### 3.1. Oxidative Stress

Oxidative stress is one of the most critical and extensively studied mechanisms of anthracycline-induced cardiac dysfunction. The iron-mediated excessive production of reactive oxygen species (ROS) in cardiomyocytes by anthracyclines renders cells vulnerable to oxidative stress, resulting in the degradation of cellular proteins, lipids, DNA, and membrane lipid peroxidation [8,9,10]. On the other hand, the dysregulation of antioxidant enzymes and pathways in cardiac and endothelial cells leads to redox imbalance and exacerbates oxidative stress. Key antioxidant enzymes—including peroxidase, catalase, and superoxide dismutase—are markedly reduced in cardiac tissue following doxorubicin exposure through mechanisms such as Nrf2 suppression and downregulation of the cAMP/PKA/SIRT1 pathway [11,12].

ROS production, antioxidant defense, and cell death pathways in cardiac cells are tightly regulated by various miRs. MiR-21 modulates key genes involved in redox homeostasis, such as SOD2 and PTEN, thereby influencing the balance between ROS generation and cardiac injury [13,14]. Chemotherapy-induced downregulation of miR-200a, together with miR-144, weakens antioxidant defenses through Nrf2 suppression and related mechanisms [15]. Similarly, miR-140-5p targets Nrf2 and Sirt2, further promoting myocardial oxidative stress [16]. Indirectly, miR-34, miR-146a, and miR-126 help to mitigate oxidative stress by suppressing anti-apoptotic proteins, reducing pro-inflammatory signaling, and enhancing angiogenesis, respectively [17,18,19,20]. Finally, miR-210 supports cardiac and endothelial cell survival by attenuating ROS levels and cellular sensitivity to oxidative injury [21,22].

### 3.2. Disruption of Mitochondrial Function

Mitochondria are the primary organelles responsible for ROS production, and their dysfunction is a major contributor to oxidative stress. They are abundant in cardiomyocytes and represent a key target of anthracyclines. As anthracyclines accumulate within cardiac mitochondria, they interact with iron and cardiolipin, leading to excessive ROS generation and mitochondrial dysregulation [23,24,25]. By targeting genes that are involved in mitochondrial biogenesis and function, miRs participate in mitochondrial regulation. For example, miR-181c regulates the expression of mitochondrial genes, such as mt-COX1, leading to mitochondrial ROS production [26]. MiR-155 regulates mitochondrial damage via the Nrf2/HO-1 signaling pathway, affecting the high glucose-induced cardiac fibrosis [27]. Furthermore, miR-30 family members are reduced as a response to doxorubicin treatment, disrupting mitochondrial integrity and increasing apoptosis [28].

MiR-130a exacerbates FUNDC1-mediated mitochondrial fission while suppressing mitochondrial fusion via MFN1/MFN2, leading to fragmented mitochondria and ROS accumulation [29,30]. Beyond chemotherapy-induced stress, miR-130a also amplifies mitochondrial injury in ischemia–reperfusion models [30,31]. Targeted inhibition of miR-130a may therefore represent a potential therapeutic strategy for CTRCD. Moreover, mitochondrial homeostasis is closely linked to apoptosis, and several miRs—including miR-423 and miR-499—regulate pro-apoptotic signaling through pathways such as Wnt/β-catenin and p53 [32,33].

### 3.3. Inhibition of Topoisomerase 2 Beta (Top2β)

The inhibitory effect of anthracyclines on Top2β has been extensively studied as a key mechanism of anthracycline-induced cardiotoxicity. When inhibited by anthracyclines, Top2β promotes DNA intercalation and double-strand breaks in cardiomyocytes, leading to apoptosis and mitochondrial dysfunction [34,35,36]. While this pathway is primarily associated with cancer resistance, it also serves as an indicator of cardiotoxicity risk. Notably, miR-9 appears to reduce TOP2B expression and may contribute to chemotherapy resistance [37].

### 3.4. Abnormal Iron (Fe) Metabolism

Mitochondria are the primary intracellular sites of iron (Fe) storage and regulation. A family of mitochondrial iron transporters, including MFRN1 and MFRN2, controls the transfer of Fe into the mitochondria [38]. Minotti et al. demonstrated that a metabolite of doxorubicin binds the Fe–sulfur complex of IRP-1, inducing Fe depletion. Concurrently, ROS accumulation inactivates IRP-2, promoting the binding of IRPs to iron regulatory elements, which ultimately leads to intracellular Fe accumulation [39]. Conversely, the mitochondrial exporter ABCB8 helps maintain Fe balance by facilitating its export from mitochondria. Doxorubicin disrupts this process by reducing ABCB8 protein levels in cardiomyocytes, thereby increasing mitochondrial Fe content [24,40]. Collectively, these alterations drive Fe overload, mitochondrial dysfunction, and oxidative stress.

Among the miRs involved in Fe homeostasis, miR-210 and miR-34a are the most significant. Fe deficiency and hypoxia activate miR-210 expression, positioning it as a regulator of Fe metabolism. MiR-210 decreases transferrin receptor 1 and iron-sulfur cluster scaffold protein expression, thereby influencing Fe uptake and mitochondrial function [41]. This regulatory action prevents Fe overload and subsequent cell toxicity. In contrast, miR-34a has been linked to Fe-mediated oxidative injury induced by anthracyclines [18]. Modulating these miRs, or restoring Fe balance, may represent promising strategies for cardioprotection against anthracycline-induced toxicity.

### 3.5. Apoptosis

Multiple pathways contribute to anthracycline-induced cardiomyocyte apoptosis. Both intrinsic and extrinsic apoptotic cascades are activated in response to anthracyclines. The intrinsic pathway is primarily regulated by the anti-apoptotic Bcl-2 protein family, which modulates Bax/Bak and caspase-3 activity. This leads to mitochondrial outer membrane permeabilization and subsequent cytochrome c release [42]. The p53 pathway, also part of intrinsic apoptosis, is strongly upregulated by doxorubicin [43,44]. In contrast, the extrinsic pathway is mediated by death receptors such as Fas, TNF-α, and TRAIL, as well as caspase-8 and -10 [44,45,46]. Additional signaling cascades, including PI3K/AKT/mTOR, ASK1, and p53, further contribute to apoptosis and autophagy regulation [47,48].

Several miRs are involved in modulating these apoptotic pathways by targeting pro- and anti-apoptotic proteins. For example, miR-21, miR-1, and miR-499 regulate caspase-9 in cancer cells, Bcl-2 in cardiomyocytes, and apoptosis-related genes in breast cancer, respectively [44]. Let-7b-5p mimics enhance apoptosis by upregulating Bcl-2 family members in keratinocyte cells [49], while miR-29b directly targets the pro-apoptotic protein Bax [50]. Similarly, reduced let-7g expression in doxorubicin-treated models downregulates Bcl-xl, thereby promoting apoptosis [51]. Let-7a suppresses pro-apoptotic genes, such as Fas ligand and Bax, in chemotherapy-treated ovarian cells [52]. In addition, miR-130a upregulation increases caspase-3 activation, thereby driving extrinsic apoptosis, while simultaneously reducing PPARγ-mediated apoptosis and inflammation [29]. Conversely, miR-133b and miR-146a exert protective effects against anthracycline-induced cardiotoxicity by reducing cardiomyocyte apoptosis and dampening pro-inflammatory signaling [53].

### 3.6. Fibrosis

Myocardial fibrosis is a common mechanism underlying CTRCD, driven by chronic inflammation, structural remodeling, and the disruption of the normal myocardial architecture. Activated cardiac fibroblasts promote excessive deposition of collagen and other extracellular matrix proteins in the myocardium, as a consequence of oxidative stress, mitochondrial dysfunction, calcium dysregulation, and apoptosis [54,55,56]. Matrix metalloproteases (MMPs), such as MMP-1 and MMP-2 are also impaired by anthracyclines [57]. In addition, pro-inflammatory cytokines (e.g., interleukin-6 and TNF-α), together with TGF-β and SMAD3 signaling, are upregulated following chemotherapy exposure and play key roles in fibrosis progression [58].

MiRs regulate chemotherapy-induced fibrosis through multiple pathways. For instance, miR-29b is upregulated in plasma after anthracycline treatment and has been implicated in extracellular matrix remodeling and fibrotic changes [59]. Similarly, miR-29a modulates collagen synthesis and correlates with troponin T levels [60]. In contrast, miR-155 promotes high glucose–induced cardiac fibrosis by modulating the Nrf2/HO-1 signaling pathway [27]. Upregulation of miR-21 in response to acute myocardial injury enhances PTEN inhibition and MMP-2 expression, thereby driving fibroblast proliferation and myocardial fibrosis [61]. MiR-34a also contributes to fibrosis by inhibiting SIRT1 and other cardioprotective factors against remodeling [62]. Finally, miR-126 and miR-423 have been linked to fibrotic changes through their roles in vascular injury and myocardial remodeling, respectively [60].

## 4. Association of miRs with CTRCD

The most common miRs associated with CTRCD are Let-7f, miR-1, miR-29, miR-34a, miR-126, miR-130a, miR-133, miR-30e and miR-140-5p, miR-320, and miR-499. Table 2 summarizes clinical trials in cancer patients that identified miRs with potential roles as biomarkers of CTRCD. However, the definition of cardiotoxicity varies across these studies, with some including subclinical markers such as troponin elevation or myocardial strain abnormalities, underscoring the complexity of diagnosing early cardiac dysfunction.

### 4.1. Let-7 Family

In human, let-7a, b, c, d, e, f, g, i, miR-98 and miR-202 are members of the let-7 family [79]. Some of them are differentiated after doxorubicin therapy and in cardiac impairment and fibrosis [80,81,82,83]. Let-7f and let-7g appear to be particularly involved in chemotherapy-induced cardiotoxicity. In a study of 363 breast cancer patients treated with anthracyclines and cyclophosphamide followed by docetaxel as neoadjuvant therapy, baseline let-7f levels were associated with subsequent cardiac impairment and showed a negative correlation with other cardiac biomarkers [66]. Similarly, in 179 patients with triple-negative breast cancer who received the same regimen, let-7f expression demonstrated prognostic value for a lower risk of cardiotoxicity [67]. In preclinical models, let-7g was significantly downregulated in rat heart tissue following exposure to 18 mg/kg doxorubicin, and its levels correlated with troponin T, heart rate, and pulse pressure [51].

### 4.2. miR-1 Clusters

MiR-1 and miR-133 form two clusters essential for skeletal and cardiac muscle development and function and are strongly linked to acute myocardial infarction [84,85,86], but the results regarding their contribution in early detection of cardiotoxicity are controversial. Within 24 h of anthracycline infusion, miR-1 upregulation showed a stronger correlation with cardiotoxicity than troponin I [65,87]. Leger et al. reported similar findings, with miR-1 elevation detected as early as 6 h after anthracycline exposure and persisting for up to 18 h [59]. Both miR-1 and miR-133 were upregulated at 3 and 6 months, correlating positively with troponins I and T, in 17 breast cancer patients receiving chemotherapy and trastuzumab [73]. Conversely, in a follow-up of more than 5 years, Totoń-Żurańska et al. found reduced miR-1 levels in cancer survivors compared with healthy controls [86]. Importantly, miR-133a levels allowed discrimination of cardiotoxicity in breast cancer patients treated with doxorubicin [68].

In mouse models, miR-1 was upregulated within 2 h of doxorubicin infusion in animals with reduced ejection fraction [87]. Findings on miR-133b are inconsistent: two studies in rats and cardiomyocytes reported downregulation [88,89], whereas breast cancer patients showed increased expression during doxorubicin treatment [65]. Functionally, miR-133b overexpression reduces fibrosis and limits apoptosis and hypertrophy in cardiomyocytes [88,90]. Similarly, miR-133a regulates proliferation, hypertrophy, and survival, positioning both miR-133a and miR-133b as potential biomarkers and therapeutic targets of cardiotoxicity.

### 4.3. miR-29 Family

The miR-29 family, particularly miR-29b, plays a pivotal role in cardiac remodeling, fibrosis, and apoptosis and is closely associated with CTRCD. In pediatric and young adult patients, miR-29b was the second miR upregulated within 6 h after anthracycline treatment, correlating with both anthracycline dose and high-sensitivity troponin T levels [59]. Similarly, increased miR-29a levels correlated with elevated cardiac troponin I 6 months after initiation of doxorubicin therapy. By contrast, in doxorubicin-treated rats, myocardial miR-29b was downregulated, promoting apoptosis and cardiac dysfunction [50]. Mechanistically, miR-29b regulates multiple collagens and extracellular matrix genes [91] and its downregulation may control remodeling and fibrosis after CTRCD [92]. Elevated miR-29a levels in patients with hypertrophic cardiomyopathy further support its role in cardiac fibrosis and matrix regulation—processes also relevant to CTRCD [93,94].

### 4.4. miR-30 Family

The miR-30 family consists of miR-30a, miR-30b, miR-30c, miR-30d, and miR-30e, and its role in CTRCD is well documented, particularly in doxorubicin exposure. In both in vivo and in vitro models, miR-30a, miR-30d, and miR-30e were downregulated through GATA-6, leading to cardiac toxicity. Overexpression of miR-30 family members reduced apoptosis and ROS levels in cardiomyocytes, underscoring their cardioprotective function [89,95,96]. Loss of miR-30 diminishes its protective effects, such as suppression of pro-apoptotic signaling, regulation of β-adrenergic pathways, and preservation of mitochondrial integrity [95,97]. Clinically, Zhou et al. observed a gradual increase in circulating miR-30c in 80 non-small cell lung cancer patients receiving chemotherapy plus bevacizumab. Elevated levels correlated with cardiotoxicity during treatment, but not after therapy completion [72].

### 4.5. miR-34a

MiR-34a expression rises significantly in the blood of breast cancer patients after initial doxorubicin administration and remains elevated even one year after treatment completion [70,75,78]. It is noteworthy that the study by Pierre Frères et al. employs a distinct sample collection timepoint compared to other clinical studies, with measurements taken approximately 8 days prior to surgery, and this timing varies individually for each patient [78]. Lakhani et al. reported similar findings in HER2-positive breast cancer patients, where miR-34a upregulation correlated with troponin I elevation during therapy [60]. A striking increase in miR-34a was also observed after two cycles of epirubicin [62]. Preclinical studies link miR-34a to early cardiac injury, although it does not consistently reflect changes in left ventricular ejection fraction [60,78].

### 4.6. miR-126

MiR-126 is one of the most extensively studied miRs, with established roles in inflammation, angiogenesis, carcinogenesis, and metastasis [98]. Reduced plasma levels of miR-126 have been documented in cardiovascular conditions including acute myocardial infarction, chronic kidney disease–related endothelial dysfunction, and stroke, supporting its prognostic value [99,100,101,102]. In cardiotoxicity, baseline miR-126 levels were significantly lower in breast cancer patients who developed cardiotoxicity following neoadjuvant epirubicin therapy, and negatively correlated with troponin I expression [66]. Conversely, high baseline miR-126 levels predicted lower risk of cardiac impairment in patients with resectable triple-negative breast cancer [67]. Lakhani et al. further reported gradual miR-126 upregulation correlating with both troponin I and T during chemotherapy [60].

### 4.7. miR-130a

MiR-130a, highly expressed in cardiac tissue, regulates development and apoptosis [103,104]. In a cohort of 72 breast cancer patients treated with anthracyclines and trastuzumab, 17% developed cardiotoxicity within 15 months, reflected by elevated baseline and treatment-associated miR-130a levels [64]. Preclinical studies suggest that miR-130a downregulation may serve as a therapeutic strategy against doxorubicin-induced cardiac injury [29].

### 4.8. miR-140

MiR-140-3p is one of the miRs upregulated in doxorubicin-treated breast cancer patients with abnormal LVEF [70]. MiR-140-5p is also increased in vivo and in vitro models after doxorubicin exposure by direct regulation of oxidative stress through Nrf2 and Sirt2 [16,105]. It is also involved in bevacizumab-mediated cardiotoxicity in cardiomyocytes targeting VEGFA signal pathway [106].

### 4.9. miR-320a

MiR-320a is the family member most strongly associated with cardiotoxicity. In doxorubicin-treated hearts, it regulates cardiotoxic responses through VEGFA signaling, similarly to miR-140-5p. In contrast, circulating miR-320a was reduced in chemotherapy patients, including those receiving anthracyclines. Its regulation also influences cardiac microvascular density and endothelial function by modulating proliferation and apoptosis [107].

### 4.10. miR-499

MiR-499 increases within 2 h of doxorubicin treatment in mice with impaired cardiac function. In young patients, it is among the earliest and most dose-dependent miRNAs elevated following anthracycline therapy [59]. In breast cancer patients, miR-499 upregulation at 6 months post-doxorubicin initiation correlated with elevated cardiac troponin T [60]. Conversely, in cardiomyocytes and animal models, miR-499-5p was downregulated, and its restoration protected against chemotherapy-induced cardiac damage by regulating p21 and inhibiting apoptosis [32].

## 5. Discrepancies in miR Expression Patterns

**Figure 1:**
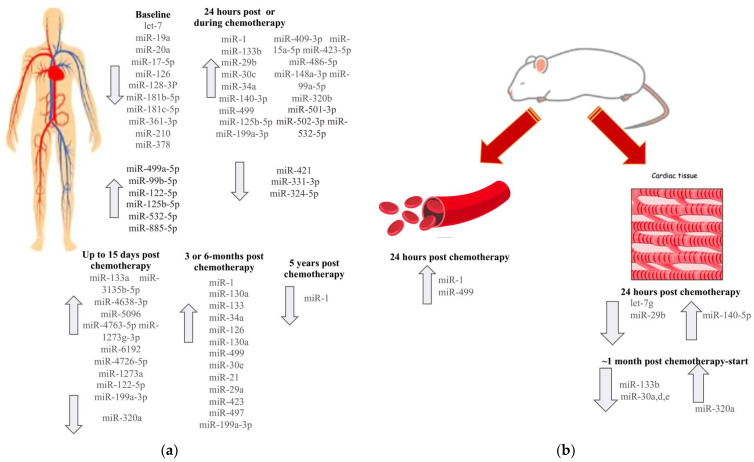
Expression of CTRCD-related miRs at different timepoints of chemotherapy in human blood (**a**) and in mice models (**b**). (**a**): the miRs are categorized based on its temporal measurement: at baseline prior to chemotherapy; 24 h post or during chemotherapy; up to 15 days post chemotherapy; 3 to 6 months post chemotherapy; and 5 years post chemotherapy. Upward arrows indicate upregulated miRs, while downward arrows indicate downregulated miRs at each timepoint. (**b**): Chemotherapy administration is followed by sampling of circulating blood and cardiac tissue at defined intervals to assess miR expression changes. The arrows indicate key miRs whose expression is upregulated or downregulated in blood plasma and cardiac tissue following chemotherapy at 24 h and approximately 1 month after.

For several miRs, we observed discrepancies between tissue and blood expression. For instance, while miR-29 and miR-499 are downregulated in cardiomyocytes following doxorubicin exposure, their elevated serum levels may reflect release from damaged cardiac cells into the circulation [32,50,59,60]. Conversely, miR-320a was reduced in the circulation of patients with acute myelogenous leukemia after doxorubicin treatment, but upregulated in the hearts and cardiomyocytes of doxorubicin-treated mice [107]. Figure 1 illustrates these divergent expression patterns, highlighting the contrasting dynamics of circulating versus tissue-specific miRNAs in both human and murine models, and underscoring their potential as circulating biomarkers for monitoring cardiotoxicity.

## 6. Cardiotoxicity and Correlated miRs with Other Anticancer Regimes

Beyond anthracyclines, several other anticancer agents, including trastuzumab, bevacizumab, cyclosporin A, and fluorouracil (5-FU), can also induce cardiotoxicity. Among these, cardiac impairment is one of the most frequent adverse effects of 5-FU, underscoring the importance of identifying potential therapeutic biomarkers. In cardiomyocytes, 5-FU reduces miR-199-5p expression, leading to cardiac injury, whereas restoring miR-199-5p levels mitigates 5-FU–induced cardiotoxicity [108]. Similarly, miR-377 is overexpressed in cyclosporin A–treated cardiomyocytes, promoting apoptosis [99]. Upregulation of miR-1254 and miR-579 has been strongly correlated with symptomatic bevacizumab-induced cardiotoxicity, independent of acute myocardial infarction [71]. In addition, Nourmohammadi et al. reported downregulation of miR-29 and miR-30b-5p in cyclosporin A–treated rats compared with controls, linking this dysregulation to progressive fibrosis and hypertrophy [109].

## 7. Clinical Implication

Early detection, risk stratification, and potential therapeutic use are the most important clinical roles of miRs in CTRCD. It is well-investigated that elevations in troponin detected shortly after chemotherapy administration reflect acute and direct myocardial cell injury or necrosis and correlate with later reductions in LVEF [110,111,112]. On the contrary, B-type natriuretic peptide (BNP) levels tend to rise more gradually and remain elevated during and after chemotherapy, indicating ongoing or chronic cardiac dysfunction and heart failure and poorer cardiac outcomes over the longer term [112,113,114].

Several miRNAs, including miR-34a, miR-29b, miR-1, miR-133a, and miR-499, show consistent early upregulation after anthracycline exposure, correlating with conventional biomarkers and LVEF reduction [32,50,59,60,65,68,78]. MiR-423, in particular, has strong predictive value for cardiac injury and heart failure [78,115,116]. Risk stratification can also be guided by baseline miRNA levels: let-7f, miR-126, and miR-130a have demonstrated predictive value for cardiotoxicity risk [64,66]. Both miR-130a and miR-29a exhibit high sensitivity for detecting cardiac remodeling after chemotherapy or anti-HER2 therapy [64,78]. Therapeutically, modulation of miRNAs offers a promising strategy. Preclinical studies suggest that targeting specific miRNAs—such as miR-1, miR-30e, miR-34a, let-7, miR-21, miR-130a, and miR-499—can attenuate chemotherapy-induced cardiotoxicity [32,62,117,118,119].

Overall, circulating miRNAs represent minimally invasive and sensitive biomarkers for the early detection of subclinical CTRCD, enabling the timely identification of patients at risk before overt cardiac dysfunction occurs. Integrating longitudinal miRNA profiling with established biomarkers and cardiac imaging could provide a more comprehensive and precise risk assessment. Moreover, machine learning and systems biology approaches hold potential for defining unique miRNA expression patterns or signatures specific to CTRCD.

## 8. Conclusions

Several miRNAs show strong potential as biomarkers for addressing the unmet need for the early detection of CTRCD. The diverse pathways leading to CTRCD highlight the importance of differential miRNA regulation. Among the most frequently implicated are let-7f, miR-1, miR-29, miR-34a, miR-126, miR-130a, and miR-133. Standardization of detection methods and validation in larger, multicenter cohorts will be essential for clinical translation. Ultimately, combining multiple miRNAs into a biomarker panel may provide the most effective prognostic tool for early detection of CTRCD and timely implementation of cardioprotective strategies.

## Figures and Tables

**Table 1:** The correlation of the pathophysiologic mechanisms of CTRCD and miRs.

miR	Mechanism of CTRCD	Regulation	Associated Pathways
miR-1	Oxidative stress and endogenous apoptosis	Upregulation	Bcl-2 in cardiomyocytes
let-7 (a, b, f, g)	Apoptosis and fibrosis	Down- or upregulation	BCL2 family members expression and caspase-3 activity
miR-21	Oxidative stress and endogenous apoptosis	Upregulation	Targeting redox genes such as SOD2, PTEN, caspase-9
miR-29b	Apoptosis and fibrosis, extracellular matrix remodeling	Upregulation	Pro-apoptotic Bax and collagen protein
miR-30	Oxidative stress, mitochondrial function and apoptosis	Downregulation	GATA-6 and other pro-apoptotic proteins
miR-34a	Apoptosis, Fe-mediated oxidative stress and fibrosis	Upregulation	Regulation of SIRT1
miR-126	Angiogenesis and fibrosis	Downregulation	Regulation of VEGF inhibitor
miR-130a	Mitochondrial dysfunction, oxidative stress, exogenous apoptosis and inflammation	Upregulation	Increase in mitochondrial fission and caspase-3 activation, reduce of mitochondrial fusion
miR-133a/b	Apoptosis and fibrosis	Down- or Upregulation	Pro-inflammatory signals
miR-140-5p	Oxidative stress, VEGFA pathway	Upregulation	Targeting Nrf2 and Sirt2
miR-144	Oxidative stress	Downregulation	Nrf2 suppression
miR-146a	Apoptosis and fibrosis	Up- or downregulation	Regulation of pro-inflammatory proteins
miR-200a	Oxidative stress	Downregulation	Nrf2 suppression
miR-210	Oxidative stress, Fe homeostasis, apoptosis and angiogenesis	Downregulation	Targeting caspase-3, caspase-8, AIFM3, VEGF, HGF
miR-423	Apoptosis and oxidative stress	Upregulation	Activation of caspases
miR-499	Mitochondrial dysfunction, apoptosis and fibrosis	Up- or downregulation	P21 pathway

AIFM3: Apoptosis-inducing factor mitochondria-associated 3, Bax: BCL2 Associated X, Bcl-2: B-cell lymphoma-2, HGF: Hepatocyte growth factor, Nrf2: Nuclear factor erythroid 2-related factor 2, PTEN: Phosphatase and tensin homolog, SIRT: Sirtuin, SOD2: Superoxide dismutase 2, VEGF: Vascular Endothelial Growth Factor.

**Table 2:** Clinical trials of CTRCD-related miRs in cancer patients.

Time of Blood Samples Collection	Chemotherapy Regimens	N of Patients (N with Cardiotoxicity)	miR	Expression Correlated with Cardiotoxicity	Reference
**During chemotherapy**					
At baseline, after 2 weeks of neo-adjuvant anti-HER2 therapy, and immediately before surgery	Trastuzumab + Paclitaxel or Lapatinib + Paclitaxel or Trastuzumab + Lapatinib + Paclitaxel → surgery → FECx3	9 (9)	At 2 weeks interval: miR-125b-5p, miR-409-3p, miR-15a-5p, miR-423-5p, miR-148a-3p, miR-99a-5p, and miR-320b	Upregulation	[63]
Every 3 months throughout the 15-month of adjuvant treatment for HER-2 positive breast cancer	EC-D + Trastuzumab	72 (12)	miR-130a	Upregulation	[64]
Baseline, 3 weeks after each cycle of doxorubicin	Doxorubicin + cyclophosphamide every 3 weeks for 4 cycles followed by paclitaxel for 12 weeks or docetaxel every 3 weeks	56 (10)	miR-1	Upregulation	[65]
Baseline	EC-D neoadjuvant chemotherapy	363 (19)	let-7f, miR-17-5p, miR-20a, miR-126, miR-210 and miR-378	Downregulation	[66]
Before and after a cycle of chemotherapy (6, 12 and 24 h)	24 patients AC, 9 patients non-anthracycline-chemotherapy	33	miR-1, miR-29b, miR-499	Upregulation	[59]
Baseline	EC-D	179 (9)	let-7f, miR-19a, miR-20a, miR-126, and miR-210	Downregulation	[67]
Baseline and up to 7 days after the completion of chemotherapy	All patients received doxorubicin. 4 patients with HER2+ cancer and 2 patients with TNBC in each group	12 (6)	miR-133a	Upregulation	[68]
Baseline and 4-cycle treatment	4 cycles of anthracycline (one every 21 days)	10 (5)	miR-122-5p	Upregulation	[69]
Baseline and after the first cycle of chemotherapy	AC	20 (8)	miR-140-3p, miR-486-5p, miR-501-3p, miR-502-3p, miR-532-5p/miR-421 miR-331-3p, miR-324-5p	Upregulation/Downregulation	[70]
Within 24 h since the onset of symptoms of CHF in comparison with control patients	Bevacizumab	178 (88)	miR-1254, miR-579	Upregulation	[71]
Before, during (at 2, 4 and 8 weeks) and 1 month after chemotherapy	Bevacizumab + chemotherapy	80 (30)	miR-30c	Upregulation	[72]
**Long-term follow up**					
At baseline, 3 and 6 months post trastuzumab therapy	Trastuzumab ± pertuzumab + Paclitaxel or Docetaxel ± carboplatin	17 (3)	miR-34a, miR-21, miR-133, miR-1, miR-30e	Upregulation	[73]
At baseline, 3 and 6 months post doxorubicin therapy	Doxorubicin	17 (4)	miR-423, miR-499, miR-126, miR-29a, miR-34a	Upregulation	[60]
Baseline and different intervals after completion of treatment	TAC or AC	20 (10) in main cohort and 32 (7) in validation cohort	miR-4732-3p	Downregulation	[74]
Baseline and at 1, 3, 6 and 12 months after the end of chemotherapy	Doxorubicin or epirubicin	88 (30)	At baseline: miR-499a-5p, miR-99b-5p, miR-122-5p, miR-125b-5p, miR-532-5p and miR-885-5p/miR-128-3p, miR-181b-5p, miR-181c-5p and miR-361-3p	Upregulation/Downregulation	[75]
After adjuvant chemotherapy	Anthracycline-based regimen, followed by paclitaxel or docetaxel	70 (33)	miR-3135b-5p	Upregulation	[76]
Before and after chemotherapy	Anthracyclines	19 (5)	miR-4638-3p, miR-5096, miR-4763-5p, miR-1273 g-3p, miR-6192, miR-4726-5p, and miR-1273a	Upregulation	[77]
Baseline, after 2 cycles, 8 days before surgery and 3 months later	Neoadjuvant 4 cycles EC → Paclitaxel 12 weeks 17 patients with HER2-positive cancer received trastuzumab or lapatinib	45 (7)	miR-126-3p, miR-199a-3p, miR-423-5p, miR-34a-5p	Upregulation	[78]

AC: Adriamycin and cyclophosphamide, CHF: Cognitive heart failure, D: Docetaxel, EC: Epirubicin and cyclophosphamide, FEC: Fluorouracil, epirubicin and cyclophosphamide, HER2: Human epidermal growth factor receptor 2, TAC: Docetaxel, adriamycin and cyclophosphamide, TC: Docetaxel and cyclophosphamide, TNBC: Triple negative breast cancer.

## Data Availability

Data sharing is not applicable to this article as no new data were created or analyzed.

## Abbreviations

The following abbreviations are used in this manuscript:

5-FU	Fluorouracil
ABCB8	ATP-binding cassette sub-family B member 8
AIFM3	Apoptosis-inducing factor, mitochondria-associated 3
AKT	Protein kinase B
ASK1	Apoptosis Signal-regulating Kinase 1
Bak	BCL2 Antagonist/Killer 1
Bax	BCL2 Associated X
Bcl-2	B-cell lymphoma-2
BNP	B-type natriuretic peptide
CTRCD	Chemotherapy-related cardiac dysfunction
cAMP	Cyclic adenosine monophosphate
DNA	Deoxyribonucleic acid
miRs	MicroRNAs
miR	MicroRNA
FUNDC1	FUN14 Domain Containing 1
HER2	Human epidermal growth factor receptor 2
HGF	Hepatocyte growth factor
HO-1	Hyperoxide ion
IRP	Iron regulatory protein
LVEF	Left ventricular ejection fraction
MFN	Mitofusin
MFRN	Mitoferrin
MMPs	Matrix metalloproteases
mTOR	mechanistic target of rapamycin
Nrf2	Nuclear factor erythroid 2-related factor 2
NSCLC	Non-small cell lung cancer
PI3K	phosphatidylinositol 3-kinase
PKA	Protein kinase A
PPARγ	Peroxisome proliferator-activated receptor gamma
PTEN	Phosphatase and tensin homolog
RNA	Ribonucleic acid
ROS	Reactive oxygen species
SIRT	Sirtuin
SMAD3	Mothers against decapentaplegic homolog 3
SOD2	Superoxide dismutase 2
TGF	Transforming growth factor
TNF	Tumor necrosis factor
Top2β	Topoisomerase 2 beta
TRAIL	TNF-related apoptosis inducing ligand
VEGF	Vascular Endothelial Growth Factor
